# Physiotherapy management of Long-COVID: an evidence-based approach

**DOI:** 10.1016/j.bjpt.2026.101609

**Published:** 2026-06-19

**Authors:** Jack Reeves, Enya Daynes, Tania Janaudis-Ferreira, Kriti Agarwal, Lissa Spencer, Ling-Ling Tsai, Jennifer A Alison

**Affiliations:** a Discipline of Physiotherapy, Graduate School of Health, Faculty of Health, University of Technology Sydney, NSW, 2008, Sydney, Australia; b Department of Physiotherapy, Royal Prince Alfred Hospital, Sydney Local Health District, NSW, 2050, Sydney, Australia; c Sydney School of Health Sciences, Faculty of Medicine and Health, The University of Sydney, Sydney, Australia; d Centre for Exercise and Rehabilitation Science, Biomedical Research Centre–Respiratory, University Hospitals of Leicester NHS Trust, Leicester, LE3 9QP, UK; e Respiratory Epidemiology and Clinical Research Unit, Centre for Outcomes Research and Evaluation, Research Institute of McGill University Health Centre (RI MUHC), Montreal, QC, Canada; f School of Physical and Occupational Therapy, Faculty of Medicine and Health Sciences, McGill University, Montreal, QC, Canada

**Keywords:** Long-covid, Physical therapy, Post-covid condition, Post-acute sequelae of covid

## Abstract

**Background:**

Long-COVID is a heterogenous, episodic, and multisystemic condition which can result following infection with a novel pathogen, severe acute respiratory syndrome coronavirus-2 (SARS-CoV-2). Whilst a precise pathophysiological cause is unknown, several mechanisms are hypothesised, each with plausible scientific rationale. Most people experiencing persistent symptoms following COVID-19 infection recover, yet some experience severe and debilitating illness for years after infection. Strategies to manage the sequelae of Long-COVID can improve the lives of sufferers.

**Objective:**

To describe the physiotherapy management of Long-COVID based on current evidence.

**Content:**

Those with ‘invisible illness’ (illness without outwardly visible signs), such as Long-COVID, often report stigmatisation and scepticism from healthcare systems. Validation of the experience of those with Long-COVID is therefore crucial to ensure patient-centred care. Thorough patient assessment is required to provide tailored management approaches given the diversity of Long-COVID presentations. Red flags that may contraindicate certain rehabilitation approaches (particularly exercise-based interventions) or that warrant further investigation should be considered. Assessments of fatigue, post-exertional malaise, respiratory symptoms, neurocognitive symptoms (i.e., brain fog), physical function, and orthostatic intolerance are strongly recommended. Management strategies may involve pacing and energy conservation techniques, pulmonary rehabilitation, inspiratory muscle training, dysfunctional breathing retraining, lifestyle and dietary strategies to manage orthostatic intolerance, and return-to-work planning.

**Conclusion:**

Physiotherapists are well positioned to deliver individualised, patient-centred, and validating care based on best available evidence.

## Highlights

• Long-COVID requires individualised, patient-centred physiotherapy care.
• Screen for red flags and post-exertional malaise before prescribing exercise.
• Pacing and energy conservation are useful for fatigue management.
• Multidisciplinary care supports recovery and return-to-work planning.

## Background

### Rationale

On the 5th of May 2023, the Director-General of the World Health Organisation (WHO) declared an end to the global health emergency caused by severe acute respiratory syndrome coronavirus-2 (SARS-CoV-2), abbreviated to COVID-19.^1^ This was a positive milestone born from international efforts to curb the virus, largely through the rapid development and introduction of vaccines which reduced the spread and severity of acute illness^2^ thereby alleviating pressures on healthcare systems.^3^ Despite the reduced imminent threat posed to global health, profound pandemic-associated morbidity endured offering unique healthcare challenges.

‘Long-COVID,’ and cognate term ‘Long-Haulers,’ originated through social media by those with lived experience.^4^ These individuals suffered persistent and heterogenous symptoms beyond the typical course of illness,^5^ which were yet to be described through traditional scientific channels. Today, Long-COVID is widely recognised as a novel condition, and our collective understanding of its optimal management is still emerging. The purpose of this article is to describe the physiotherapy management of Long-COVID based on current evidence.

### Terminology and definitions

Several terms have been coined for Long-COVID in medical literature and by national and international health organisations. The WHO use the term ‘Post-COVID Condition (Long-COVID)’ and define this as:

“The continuation or development of new symptoms three months after the initial SARS-CoV-2 infection, with these symptoms lasting for at least two months with no other explanation.”^6^

The US National Institute of Health (NIH) and the Australian Department of Healthcare and Ageing^7^ both use the term ‘Post-acute Sequelae of COVID.’ The UK National Institute of Health and Care Excellence (NICE) use a tiered naming system based on time from acute infection, where ‘Post-COVID Syndrome’ is most synonymous with the WHO definition.^8^ However, NICE consider persisting symptoms beyond the acute phase (4 weeks) to be ‘Long-COVID.’ Clinicians should be aware that while there are semantic and timeline differences in terminology and definitions, these terms largely refer to the same condition.

### Epidemiology

Incidence estimates of Long-COVID vary significantly across the globe (9–81%)^9^ likely owing to differences in data collection methods, vaccination status, virus strain, and geography. Regardless of these differences in incidence estimates, given the magnitude of global infections,^10^ a significant number of people worldwide experience ongoing illness following the acute phase of infection. Several predictive risk and protective factors^11^, ^12^, ^13^, ^14^ for developing Long-COVID have been identified and can be seen in Table 1.

**Table 1:** Predictive risk and protective factors for Long-COVID.

Risk Factors	Protective Factors
>5 symptoms at the time of acute illness	Antiviral therapy during acute illness (in those eligible)
Higher body mass index	Vaccination prior to infection
Female sex	
Pre-omicron variants	
Neurodivergence	
Co-morbidities	
Smoking history	
Hospitalisation with acute COVID infection	

### Symptoms

**Fig. 1:**
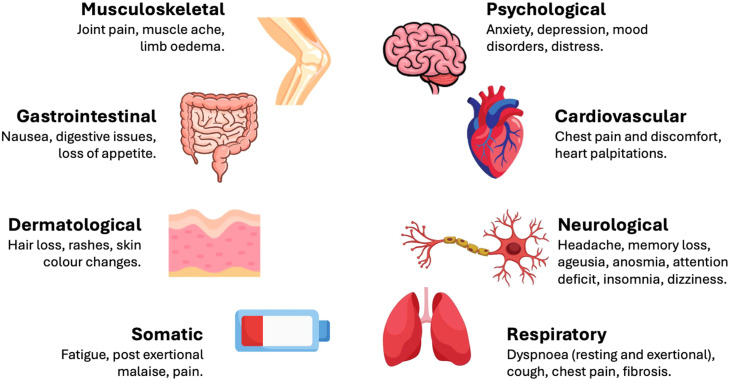
Multisystemic symptoms of Long-COVID.

Long-COVID is characterised by diverse, multisystemic symptoms which can be episodic and present variably between individuals.^15^^,^^16^ Fig. 1 demonstrates multisystemic symptoms of Long-COVID, based on a systematic review and meta-analysis.^15^ While over 200 heterogenous symptoms have been reported,^17^ with 50 of these commonly observed,^15^ those reporting Long-COVID typically present with a symptom profile centred around fatigue, dyspnoea, and ‘brain fog;’^15^ the latter being a colloquial term describing a state of temporarily diminished mental capacity to concentrate and reason.^18^ Long-COVID is often described as a relapsing-remitting condition as most experience fluctuant severity of symptoms following a bout of exertion; whether physical, cognitive, or emotional.

### Proposed mechanisms

Our scientific understanding of the pathophysiological mechanisms of Long-COVID has developed significantly in recent years, yet there remains a lack of concrete evidence as to the precise cause due to the novelty of the condition.^19^ However, key mechanisms, each with plausible scientific rationale, have been proposed based on biomarker and pathology findings in those reporting Long-COVID.^17^^,^^20^ Key hypothesised mechanisms are presented in Table 2. Whilst scientific inquiry into pathophysiological mechanisms of Long-COVID is vital for the potential development of pharmacological therapies, physiotherapy interventions typically seek to address treatable traits through applying evidence-based rehabilitation and management approaches.

**Table 2:** Key hypothesised mechanisms for Long-COVID.

Immune Dysregulation	Immune activation persists, potentially due to a reservoir of COVID virus, leading to the reactivation of latent viruses such as Epstein-Barr or other herpes viruses.^111^^,^^112^
**Microbiota Disruption**	Dysbiosis of gastrointestinal microbiome may be responsible for Long-COVID sequelae, evidenced by differences in microbiome composition, even when comparing different phenotypes of symptoms (e.g., respiratory versus neurological predominance).^113^^,^^114^
**Autoimmunity and Immune Priming**	Antibodies of a host’s immune system may mistakenly target healthy cells whose proteins resemble proteins of the COVID virus through the process of molecular mimicry, causing collateral damage to host cells.^115^
**Blood Clotting and Endothelial Abnormality**	Atypical clotting (micro and macro) has been observed at greater rates in those with COVID-related critical illness than those with non-COVID critical illness, as have endothelial abnormalities such as endotheliitis (inflammation) and atypical angiogenesis (unexpected new blood vessel formation).^116^, ^117^, ^118^, ^119^, ^120^
**Dysfunctional Neurological Signalling**	Dysautonomia (dysfunction of autonomic functions i.e., heart rate, blood pressure, body temperature), as well as other common symptoms including brain fog, insomnia, and diffuse pain, suggest signalling dysfunction in the brainstem.^115^ This is further supported with evidence of radiographical brainstem abnormalities demonstrated in people with chronic fatigue syndromes (which often share a similar symptom profile with Long-COVID).^121^

### Prognosis

Most individuals infected with COVID-19 make a full recovery. The median duration of time from infection to self-reported symptom resolution is 20 days.^21^ Even those who meet criteria for Long-COVID diagnosis can improve with time and eventually see symptom resolution.^22^ However, it is unknown if time alone will improve symptoms for all people experiencing Long-COVID, as many experience severe and debilitating symptoms years after infection. Given the recency of the COVID-19 pandemic, it is difficult to prognosticate Long-COVID symptom trajectories due to the lack of long-term data.

### Post-acute infective syndromes

Long-COVID is considered a novel condition due to its manifestation secondary to infection with a novel pathogen (SARS-CoV-2); however, it has long been reported that a small percentage of individuals experience persistent symptoms following any infection (typically viral infections). Collectively, these conditions are termed ‘post-acute infective syndromes (PAIS).’ Symptom profiles of different post-acute infective syndromes overlap significantly, with fatigue, myalgia, brain fog, and exercise intolerance typically prominent features, and therefore they likely share a similar pathology.^23^ Some examples of other PAIS are ‘post-treatment Lyme disease’ following Borrelia, ‘post-dengue fatigue syndrome’ following dengue, and ‘post chikungunya chronic inflammatory rheumatism’ following chikungunya. Long-COVID can be thought of as the post-acute infective syndrome of SARS-CoV-2.

### Myalgic encephalomyelitis/chronic fatigue syndrome (ME/CFS)

Another overlapping condition with Long-COVID is myalgic encephalomyelitis/chronic fatigue syndrome (ME/CFS).^24^ ME/CFS describes a state of severe and debilitating episodic fatigue disproportionate to exertion which may follow a viral infection.^25^^,^^26^ Assessment and management strategies for Long-COVID are often drawn from previous research into ME/CFS given their similarities and due to the recency of Long-COVID.

### Invisible illness and healthcare

Sufferers of ‘invisible illnesses’ (illness without outwardly visible signs) have a history of stigmatisation by healthcare systems. Stigmatisation may be compounded in situations where illness is not objectively measurable, as is often the case with Long-COVID,^27^ as the lack of definitive diagnostic tests may elicit scepticism to the legitimacy of symptoms presented. Those with Long-COVID seeking medical care often report dismissal of their condition by healthcare professionals.^28^ Given present-day diagnostic challenges, healthcare professionals may exhibit a reticence to acknowledge Long-COVID as a legitimate condition due to the potential for psychology to readily explain the presenting symptoms. Whilst psychology undoubtedly influences all chronic health conditions, the perpetuating deleterious narrative that Long-COVID can be attributed to psychology in isolation has the potential to worsen outcomes for those affected. Clinicians should be reminded of the growing evidence of pathophysiological mechanisms that lead to Long-COVID symptoms (see Table 2).^17^

## Assessment

Long-COVID can present heterogeneously and therefore requires thorough yet individualised assessment. Given the diversity of sequelae that can result, a multidisciplinary,^29^ patient-centred,^30^ and trauma-informed^31^ approach is recommended. Prior to considering any rehabilitation approach, particularly those that involve physical activity, clinicians should screen for red flags that require referral for further investigation. Red flags and associated reasons for concern based on advice from international health and rehabilitation organisations^32^^,^^33^ are presented in Table 3.

**Table 3:** Red flags and considerations for referral.

	**Exertional desaturation ≥3% SpO_2_**	Consider referral to a respiratory physician or general practitioner for investigation of interstitial abnormality or pulmonary dysfunction.^32^^,^^33^
	**Chest pain or inappropriate tachycardia**	Consider referral to cardiologist for investigation of myocarditis, pericarditis, or microvascular angina.^32^^,^^33^
	**Signs of autonomic dysfunction**	Consider referral to a cardiologist or general practitioner for investigation of dysautonomia (signs of disrupted homeostatic functions such as irregular or variable heart rate and blood pressure, orthostatic intolerance, and impaired thermoregulation).^32^

The assessments outlined here are not exhaustive, as additional evaluations may be warranted depending on an individual’s presentation. Rather, commonly used assessments are presented. When assessing symptoms, it is important to clarify if the symptoms were present prior to COVID-19 infection, that is, whether they are new or represent a worsening of pre-existing conditions. An overview of symptom domains and common appropriate outcome measures used can be seen in Table 4.

**Table 4:** Overview of appropriate outcome measures to assess Long-COVID symptoms.

Symptom domain	Outcome measure
Fatigue	Fatigue Severity Scale (FSS) DePaul Symptom Questionnaire – Post-exertional Malaise Short-Form (DSQ – PEM) Functional Assessment of Chronic Illness Therapy - Fatigue Scale (FACIT-Fatigue)
Respiratory (dyspnoea, cough, breathing pattern)	Modified Medical Research Council Dyspnoea Scale (mMRC) Dyspnoea-12 Borg Rating of Perceived Exertion (RPE) for Dyspnoea COPD Assessment Test (CAT) Breathing Pattern Assessment Tool Nijmegen Questionnaire Breathing Vigilance Questionnaire Capnography
Neurocognitive issues (‘brain-fog’)	Montreal Cognitive Assessment Test (MoCA) Mini-Mental State Examination (MMSE)
Function	Post-COVID Functional Scale (PCFS) 6-minute Walk Test (6MWT)* Incremental Shuttle Walk Test (ISWT)* 1-minute sit-to-stand test (1-minSTST)*
Orthostatic intolerance	NASA Lean test Tilt-table test Active Stand test Heart Rate Variability (HVR) monitoring

⁎ These assessments may cause post-exertional malaise and therefore clinicians need to exercise caution when considering their use.

### Fatigue

Fatigue is a cardinal feature of Long-COVID in many. Fatigue may involve post-exertional malaise (PEM), and related term post-exertional symptoms exacerbation (PESE), which describe the worsening of symptoms 12–48 h (or more) following a bout of exertion (whether physical, cognitive, emotional, or other) or following an activity which was previously tolerated.^34^^,^^35^ An individual’s subjective history is often enough to identify PEM, however, patient reported outcomes measures of fatigue, such as the Fatigue Severity Scale (FSS),^36^^,^^37^ may aid in characterising fatigue and assessing severity.^32^ A commonly used patient-reported measure is the DePaul Symptom Questionnaire – Post-exertional Malaise Short-Form (DSQ-PEM), a 10-item assessment of the presence and severity of PEM.^38^ In addition to patient-reported measures, physical assessments of PEM have previously been employed including two-day cardiopulmonary exercise testing (CPET). The rationale behind a two-day CPET, drawn from ME/CFS literature,^39^^,^^40^ is that those with PEM may perform reasonably on a day one CPET, however, would perform significantly worse on a day two CPET (next day) due to delayed malaise, whereas an individual without PEM can typically repeat their day one performance. Whilst this approach may objectively demonstrate an individual’s PEM to the assessor, it is likely to cause uncomfortable symptoms for the participant which may be unnecessary when PEM can be assessed subjectively.

### Respiratory symptoms

Dyspnoea, cough, chest pain, and dysfunctional breathing are commonly present in those reporting Long-COVID.^15^ Dyspnoea can be measured in real time (such as during physical activity) using the Borg Rating of Perceived Exertion for Dyspnoea Scale,^41^ or can be assessed in relation to usual physical activities using the modified Medical Research Council Dyspnoea Scale^42^ or the Dyspnoea-12.^43^ General respiratory symptoms, including breathlessness and cough, can be assessed using the COPD Assessment Test. Although originally designed for COPD, this tool may be useful in assessing respiratory symptoms post COVID-19 infection.^44^ Dysfunctional breathing, also known as a breathing pattern disorder, is a maladaptive breathing pattern alteration that causes respiratory and non-respiratory symptoms in the absence, or in excess, of underlying pathology.^45^ This can manifest as hyperventilation, thoracic-dominant breathing, or an erratic respiratory rhythm.^46^ Whilst there is no gold standard diagnostic test for dysfunctional breathing,^45^ patient reported tools such as the Nijmegen Questionnaire^47^^,^^48^ or Breathing Vigilance Questionnaire,^49^ and clinician administered tools such as the Breathing Pattern Assessment Tool,^50^ may aid in diagnosis and treatment. Capnography has also been used to assess hypocapnia due to dysfunctional breathing associated hyperventilation in people with Long-COVID.^51^

### Neurocognitive symptoms

Neurocognitive issues, often termed as ‘brain fog,’ are commonly assessed using the Montreal Cognitive Assessment Test (MoCA), a 30-point tool used to detect mild cognitive impairment by assessing domains including attention, memory, language, executive function, visuospatial skills, and orientation.^52^ However, the MoCA, along with other brief screening tests such as the Mini-Mental State Examination, may not be sensitive to all reported neurocognitive issues in those with Long-COVID^53^ and previous samples have shown normal scores with both the standard and blind versions among Long-COVID populations.^54^^,^^55^ More severe neurocognitive issues detected through these screening tests should be further assessed with advanced neuropsychological testing by a trained medical practitioner.^53^

### Function

Function can be assessed through physical testing or through patient-reported measures. Consideration should be given as to whether an individual exhibits PEM prior to the use of any physical testing, as this could exacerbate symptoms.^32^ When physical testing is deemed suitable, commonly used tests include the six-minute walk test (6MWT), the incremental shuttle walk test, and the 1-minute sit-to-stand test. Patient-reported measures, especially in the presence of PEM, are also useful to characterise function. A common condition-specific assessment is the Post-COVID Functional Scale which classifies function into five severity categories and is shown to correlate with fatigue in those with Long-COVID.^56^

### Orthostatic intolerance

Orthostatic intolerance is a broad descriptor which encompasses both orthostatic hypotension, defined as the sustained reduction in systolic blood pressure of at least 20 mmHg or diastolic blood pressure of 10 mmHg within three minutes of standing or head-up tilt to at least 60 degrees on a tilt table; and postural orthostatic tachycardia syndrome (POTS), defined as a sustained heart rate increment of ≥30 beats/minute within 10 min of standing or head-up tilt in the absence of orthostatic hypotension.^57^, ^58^, ^59^ Whilst the gold standard for assessing and diagnosing orthostatic hypotension and POTS is by tilt-table test, other cost-effective assessments can be used for screening and monitoring of symptoms clinically. These include the NASA Lean Test and the Active Stand Test, both of which involve monitoring a patient’s vital signs and symptoms while leaning or standing after a period of supine rest. Heart rate variability (HRV), which describes the variation in time intervals between consecutive heartbeats, is a non-invasive marker of orthostatic intolerance and autonomic dysfunction, and emerging evidence suggests that some individuals reporting Long-COVID may exhibit reduced HRV.^60^^,^^61^

## Management strategies

### Pacing and energy conservation

Pacing and energy conservation techniques are a useful tool in the management of long-term conditions, particularly in those who experience fatigue as a dominant symptom. Pacing and energy conservation refers to strategies that allow individuals to pace their activities to not provoke a worsening in symptoms (or “crash”). This includes planning activities over a set period (e.g., weekly) and ensuring that high energy activities are performed when energy levels are high. Pacing activities — which can include slowing down, breaking up the activity, or modifying to make it easier (e.g., ironing in sitting rather than standing) — require the individual to take a high-level view of their activities, and can be performed individually or with support of a healthcare professional. Patients who are able to adhere to pacing strategies may observe a faster recovery.^62^ The benefit of this technique is that it acknowledges variation in symptoms and supports individuals to respond appropriately to these variations. This intervention can be standalone, light touch, or integrated with other interventions, and therefore should be a key consideration for healthcare professionals; however, the evidence base for pacing/energy conservation as a standalone intervention is limited.^63^ Offering physiotherapy services with the choice of modality i.e., centre-based versus telehealth, may allow access for those with fatigue-based constraints.^64^

### Pulmonary rehabilitation

Exercise rehabilitation techniques have shown promise in the management of Long-COVID. There is a large volume of literature demonstrating the benefits of pulmonary rehabilitation (PR) for people with chronic respiratory disease, and given the overlap of some Long-COVID symptoms with those of chronic lung disease, PR could be adapted to meet the needs of a Long-COVID population.^65^ Early data began to emerge in 2021 demonstrating proof of concept for exercise-based rehabilitation in those who were hospitalised with acute COVID-19 infection and had persisting symptoms^66^ and this was soon supported by three large randomised controlled trials. Firstly, the TERECO trial demonstrated significant improvements in exercise capacity and health-related quality of life (HRQoL) following a six-week unsupervised exercise programme compared to a control group (six-minute walk distance 65 m [CI: 43–87], p < 0.01).^67^ Secondly, the REGAIN trial demonstrated significant improvements in HRQoL in a virtual, supervised, eight-week programme of exercise and psychological support (Patient Reported Outcomes Measurement System [PROMIS] score adjusted mean difference 0.03 [0.01 to 0.05], p = 0.02).^68^ Lastly, the PHOSP-R trial demonstrated significant improvements in an eight-week programme of exercise and education delivered as twice weekly supervised sessions (Incremental Shuttle Walking Test 52m [19–85m], p < 0.01) or as an unsupervised digital programme (34 m [1–66m], p = 0.047).^69^ In addition to these trials, systematic reviews have highlighted the benefits of exercise-based programmes in the management of Long-COVID, though programme content, delivery, and duration differ, which yielded different results, and were often poorly defined.^70^^,^^71^ Given the broad spectrum of Long-COVID symptoms and phenotypes, a suite of options for exercise based therapies could be advantageous. There is a bias in the data in favour of post hospitalised patients with Long-COVID, and further research on exercise-based rehabilitation for those who were not hospitalised with acute COVID-19 infection is warranted.^72^

### Graded exercise therapy

Exercise-based rehabilitation can be a topic of scrutiny, particularly in relation to Graded Exercise Therapy (GET), and PR can face similar criticism. There are distinct differences in GET and PR, notably, PR is individually prescribed and progressed following an individual holistic assessment and is modified in response to the individual’s progress and symptoms.^73^ Conversely, GET aims to progress exercise following a fixed protocol. PR also encompasses behaviour change techniques and symptom management supported by a programme of education whereas GET refers to an exercise programme alone. The major criticism of exercise therapy is the potential to cause PEM. In instances of PEM, exercise therapy may not be appropriate, however, the challenge remains in appropriately identifying these patients.^65^^,^^74^ It is advised that patients with Long-COVID are closely monitored when engaging in an exercise programme, and for it to be terminated if they demonstrate severe and debilitating PEM, until it is suitably investigated and better managed. High symptoms of fatigue alone are not a sole indication of PEM and may improve with exercise-based therapies^75^ therefore a thorough assessment of PEM is required with regular monitoring of symptoms as to not unnecessarily exclude patients from effective interventions, but to also promptly identify and act on PEM. It is important to consider Long-COVID as a heterogeneous and complex condition, where treatment benefits differ between patients, and as such, exercise-based therapy can be considered safe and effective in those with Long-COVID in the absence of PEM. Guidelines recommend against the use of GET in anyone reporting PEM.^32^

### Inspiratory muscle training

Numerous non-pharmacological interventions have been effective at managing breathlessness in chronic respiratory conditions and therefore may be applicable to those suffering from Long-COVID-related dyspnoea.^76^ One such promising intervention is Inspiratory Muscle Training (IMT). By applying resistance to inspiratory airflow, IMT challenges the respiratory muscles and elicits adaptations similar to those achieved through strength training in peripheral muscles.^77^ IMT has shown to produce clinically meaningful improvements in dyspnoea and HRQoL in those with chronic respiratory diseases such as COPD^78^ and has also been well tolerated in bronchiectasis.^79^ Given that respiratory muscle weakness predicts poor outcomes following COVID-19 infection,^80^ IMT may serve as a feasible entry point into broader rehabilitation programmes for patients with Long-COVID.^81^ A recent systematic review^82^ of seven studies evaluated respiratory muscle training in patients with Long-COVID, with protocols ranging from 3–14 sessions per week over periods of 2–12 weeks, and adherence generally defined as >70% completion of prescribed sessions. The review reported significant improvements in respiratory muscle strength, exercise capacity, and HRQoL in patients with Long-COVID.^82^ In addition, reduction in dyspnoea and improved ability to perform daily activities without excessive fatigue were consistently reported.^81^^,^^83^^,^^84^ Complementing this evidence, practice recommendation advise that in absence of PEM, IMT should be 30–50% of maximal inspiratory pressure (PImax) and should be progressed as tolerated.^85^ While, in the presence of mild or moderate PEM, IMT should be 30% of PImax and progressed as tolerated; with a frequency of 3 to 7 sessions per week. However, variability in study designs, instruments, and quality of evidence highlights that further standardised and high-quality research is required.^85^

### Dysfunctional breathing retraining

Dysfunctional breathing is increasingly recognised as a significant contributor to dyspnoea in patients with Long-COVID^86^, ^87^, ^88^ and has been associated with a high symptom burden, psychosocial distress, and reduced HRQoL across both adult and paediatric cohorts.^86^^,^^87^ Patient education is crucial, as understanding the benign but disruptive nature of dysfunctional breathing can itself reduce symptom-related anxiety.^86^ “Breathing retraining” forms the cornerstone of therapy.^86^^,^^89^ Breathing retraining programmes typically aim to restore nasal breathing, reduce reliance on accessory thoracic musculature, normalise respiratory rate and tidal volume, and progressively integrate corrected patterns into daily activity. Training often begins in semi-supine positions and advances through sitting, standing, and functional tasks.^86^ Relaxation and mindfulness strategies are also incorporated to address the strong interaction between emotional state and respiratory control.^46^^,^^90^ Digital and group-based programmes, including singing-based interventions, have also shown short-term benefit, although clinician-guided interventions appear superior to unsupervised approaches.^71^^,^^91^, ^92^, ^93^ Emerging evidence from trials in asthma and chronic respiratory disease suggests breathing retraining improves HRQoL,^92^^,^^93^ which may extend to Long-COVID.^91^

### Managing orthostatic intolerance

Studies have reported cardiovascular autonomic abnormalities in patients with Long-COVID, including POTS,^94^ orthostatic hypotension,^95^ and inappropriate sinus tachycardia, heart rate >100 resting supine).^96^^,^^97^ The prevalence of POTS and inappropriate sinus tachycardia in Long-COVID has been reported as 2–14%.^98^ Importantly, the prevalence of cardiovascular dysautonomia does not differ significantly between these hospitalised and non-hospitalised individuals, suggesting that acute disease severity is not a predictor for developing dysautonomia.^99^ Management strategies emphasise non-pharmacological measures such as withdrawal of exacerbating medications or behaviours, adequate fluid intake of (∼3 litres per day), salt supplementation (<10 *g* daily) in patients without hypertension or fluid overload, sleeping in a head-up tilt position (>10°) to promote intravascular volume, and the use of compression garments.^100^ Rehabilitation approaches such as autonomic conditioning therapy,^101^ non-upright exercise regimens, isometric training,^58^^,^^102^ and carefully titrated aerobic activity have been proposed,^58^^,^^102^, ^103^, ^104^ though they require caution given the risk of PEM in Long-COVID.^105^ WHO guidelines^106^ recommend a combination of patient education, self-management strategies, and physical exercise training when tolerated, along with environmental modifications to support daily activities.

### Return to work planning

Supporting return to work for individuals impacted by Long-COVID should be a key rehabilitation goal, given its strong links to HRQoL, financial situation, and long-term health outcomes.^106^ A collaborative, personalised, and phased approach is essential, recognising the unpredictable nature of symptoms such as PEM and fatigue.^107^ Physiotherapists should begin with a comprehensive assessment of fatigue, cognitive function, respiratory capacity, and functional tolerance. Education and self-management strategies such as energy conservation, breathing techniques, sleep hygiene, and stress management, empowers clients to actively engage their recovery and return to work process.^107^ The individualised return to work action plans should include prolonged, flexible, and phased reintegration, with close collaboration among the client, employer, and healthcare team.^106^, ^107^, ^108^ Work accommodations such as reduced hours, adjusted tasks, extended timelines, remote work options, and scheduled rest breaks can support a sustainable return to work.^3^ Environmental modifications in the workplace may also be needed based on individualised assessments.^106^, ^107^, ^108^ Educating employers about the fluctuating and often invisible nature of Long-COVID symptoms will help to ensure appropriate workplace accommodations.^107^ Ongoing monitoring is crucial, as some individuals may require continued adjustments. Ultimately, a flexible, interdisciplinary approach ensures return to work is safe, realistic, and aligned with each client’s functional abilities and recovery trajectory. The aim is a safe and lasting return to work, rather than an immediate return to pre-illness duties and workload.

### Pharmacological management

Currently, there is no universally accepted pharmacological treatment for Long-COVID. The condition presents with a wide range of symptoms and varying severity, requiring individualised approaches to care.^109^ Clinical decision-making is often informed by emerging evidence and experience with similar conditions. The Canadian Guidelines for Post-COVID-19 Condition offer conditional recommendations on several medications, all based on very low certainty of evidence.^110^ These should be applied with clinical judgement and in collaboration with the patient. Metformin, commonly used for type 2 diabetes, may have anti-inflammatory and anti-thrombotic effects. Some studies suggest early use during acute COVID-19 infection may reduce the risk or severity of Long-COVID.^110^ Antihistamines may be considered for individuals presenting with symptoms of mast cell activation syndrome, such as flushing, rashes, palpitations, and brain fog. Some patients report improved quality of life with their use.^110^ For individuals diagnosed with POTS or inappropriate sinus tachycardia, options include: ivabradine or beta-blockers for tachycardia and midodrine or pyridostigmine for dizziness, low blood pressure, or orthostatic intolerance. These medications may offer symptom relief and improve HRQoL under specialist supervision.^110^ Antiviral therapies such as paxlovid (nirmatrelvir/ ritonavir), remdesivir, or molnupiravir may be used in Long-COVID patients with new COVID-19 infections to reduce risk of severe outcomes and possibly mitigate Long-COVID symptoms.^110^ While prescribing medication falls outside the scope of physiotherapy practice, awareness of pharmacological options can enhance interdisciplinary collaboration, support understanding of patient care plans, and help manage expectations regarding symptom progression or improvement.

### Referral to other health professionals

A multidisciplinary approach can support the management of individuals with Long-COVID, particularly due to the diversity of symptoms presented. While physiotherapists play an important role, collaboration with other health professionals may be required. For example, referral to occupational therapists may be appropriate for support with fatigue management, pacing strategies, provision of devices and equipment for home, and return to work planning. Referral to a psychologist or other mental health professional may assist with anxiety, depression, or cognitive difficulties commonly reported in Long-COVID. Referral to a dietitian may allow nutritional optimisation and therefore energy management. In cases of persistent or complex symptoms, coordination with general medical practitioners and referral to specialist physicians may be appropriate.

**Fig. 2:**
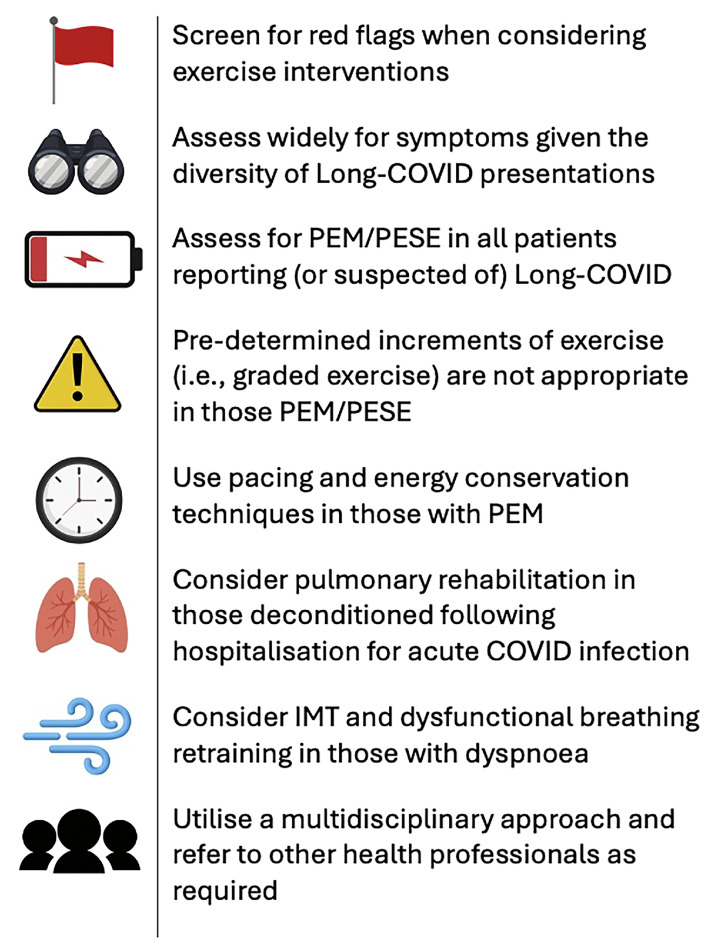
Summary of considerations for delivering physiotherapy to people with Long-COVID.

A summary figure of consideration for delivering physiotherapy to people with Long-COVID has been included (Fig. 2).

## Future direction

### Research priorities for Long-COVID management

*Long COVID Physio*, which is a patient-led organisation that seeks to advocate, educate, and provide peer support for those living with Long-COVID (https://longcovid.physio/), recently reported results of a forum aimed at identifying Long-COVID rehabilitation priorities. Based on consultation at the forum with a large (n = 794) international sample of clinicians, researchers, and people living with Long-COVID, they identified seven key research priorities.^35^ These priorities include:

1. Characterise disability
2. Identify and evaluate management and prevention strategies
3. Identify biological mechanisms
4. Standardise assessment and diagnosis
5. Describe the epidemiology
6. Describe the socioeconomic impacts
7. Explore health and disability justice

## Funding

This masterclass has been produced in-kind.

## CRediT authorship contribution statement

**Jack Reeves:** Conceptualization, Investigation, Writing – original draft, Writing – review & editing. **Enya Daynes:** Investigation, Writing – original draft, Writing – review & editing. **Tania Janaudis-Ferreira:** Investigation, Writing – original draft, Writing – review & editing. **Kriti Agarwal:** Investigation, Writing – original draft, Writing – review & editing. **Lissa Spencer:** Investigation, Writing – original draft, Writing – review & editing. **Ling-Ling Tsai:** Investigation, Writing – original draft, Writing – review & editing. **Jennifer A Alison:** Conceptualization, Investigation, Writing – review & editing.

## Declaration of competing interests

The authors have no competing interests to declare in relation to this work. All potential conflicts are listed in the submitted disclosure forms.

## References

[1] World Health Organization. (2023). Statement on the fifteenth meeting of the International Health Regulations (2005) Emergency Committee regarding the coronavirus disease (COVID-19) pandemic. World Health Organization.

[2] Feikin D.R. (2022). Duration of effectiveness of vaccines against SARS-CoV-2 infection and COVID-19 disease: results of a systematic review and meta-regression. Lancet.

[3] Thompson M.G. (2021). Effectiveness of Covid-19 vaccines in ambulatory and inpatient care settings. N Engl J Med.

[4] Callard F., Perego E. (2021). How and why patients made long covid. Soc Sci Med.

[5] Byrne A.W. (2020). Inferred duration of infectious period of SARS-CoV-2: rapid scoping review and analysis of available evidence for asymptomatic and symptomatic COVID-19 cases. BMJ Open.

[6] Soriano J.B., Murthy S., Marshall J.C., Relan P., Diaz J.V. (2022). A clinical case definition of post-COVID-19 condition by a Delphi consensus. Lancet Infect Dis.

[7] Department of Health Care and Ageing. National Post-Acute Sequelae of COVID-19 (PASC) Plan. (2024).

[8] National Institute of health and Care Excellence. National institute for health and care excellence (NICE) COVID-19 rapid guideline: managing COVID-19 (2023).

[9] Chen C. (2022). Global prevalence of post-coronavirus disease 2019 (COVID-19) condition or long COVID: a meta-analysis and systematic review. J. Infect. Dis..

[10] World Health Organisation (WHO) (2025). WHO COVID-19 dashboard. https://data.who.int/dashboards/covid19/cases.

[11] Luo D. (2024). Prevalence and risk factors for persistent symptoms after COVID-19: a systematic review and meta-analysis. Clin Microbiol Infect.

[12] Herbert C. (2025). Relationship between acute SARS-CoV-2 viral clearance and long COVID-19 (Long COVID) symptoms: a cohort study. Clin Infect Dis.

[13] Notarte K.I. (2022). Impact of COVID-19 vaccination on the risk of developing long-COVID and on existing long-COVID symptoms: a systematic review. EClinicalMedicine.

[14] Tsampasian V. (2023). Risk factors associated with post-COVID-19 condition: a systematic review and meta-analysis. JAMA Intern Med.

[15] Lopez-Leon S. (2021). >50 long-term effects of COVID-19: a systematic review and meta-analysis. Sci Rep.

[16] Brown D.A., O’Brien K.K (2021). Conceptualising long COVID as an episodic health condition. BMJ Glob Health.

[17] Davis H.E., McCorkell L., Vogel J.M., Topol E.J.L. (2023). COVID: major findings, mechanisms and recommendations. Nat Rev Microbiol.

[18] Merriam-Webster Dictionary (2021). https://www.merriam-webster.com/dictionary/slang.

[19] Gheorghita R. (2024). The knowns and unknowns of long COVID-19: from mechanisms to therapeutical approaches. Front Immunol.

[20] Lai Y.-J. (2023). Biomarkers in long COVID-19: a systematic review. Front Med (Lausanne).

[21] Oelsner E.C. (2024). Epidemiologic features of recovery from SARS-CoV-2 infection. JAMA Netw Open.

[22] Huang L. (2022). Health outcomes in people 2 years after surviving hospitalisation with COVID-19: a longitudinal cohort study. Lancet Respir Med.

[23] Choutka J., Jansari V., Hornig M., Iwasaki A. (2022). Unexplained post-acute infection syndromes. Nat Med.

[24] Sukocheva O.A. (2022). Analysis of post COVID-19 condition and its overlap with myalgic encephalomyelitis/chronic fatigue syndrome. J Adv Res.

[25] Morris G. (2019). Myalgic encephalomyelitis/chronic fatigue syndrome: from pathophysiological insights to novel therapeutic opportunities. Pharmacol. Res..

[26] Fukuda K. (1994). The chronic fatigue syndrome: a comprehensive approach to its definition and study. Ann Intern Med.

[27] Sneller M.C. (2022). A longitudinal study of COVID-19 sequelae and immunity: baseline findings. Ann Intern Med.

[28] Au L., Capotescu C., Eyal G., Finestone G. (2022). Long covid and medical gaslighting: dismissal, delayed diagnosis, and deferred treatment. SSM-Qualitative Research in Health.

[29] National Health Service (NHS). The NHS plan for improving long COVID services. (ed. (NHS), N.H.S.) (2022).

[30] Mira J.J. (2021). Proposals for person-centred care in the COVID-19 era. delphi study. Health expectations.

[31] Barton C. (2024). COVID-19 and collective trauma: implementing a trauma-informed model of care for post-COVID patients. J Adv Nurs.

[32] World Physiotherapy. World physiotherapy response to COVID-19 briefing paper 9. Safe rehabilitation approaches for people living with long COVID: physical activity and exercise. (2021).

[33] National Institute of Health and Care Excellence (2024).

[34] Bateman L. (2021). Myalgic encephalomyelitis/chronic fatigue syndrome: essentials of diagnosis and management. Mayo Clin Proc.

[35] McDuff K. (2025). Priorities for research, education, clinical practice, and policy from the Long COVID physio international forum. Cardiopulm Phys Ther J.

[36] Krupp L.B., LaRocca N.G., Muir-Nash J., Steinberg A.D. (1989). The fatigue severity scale: application to patients with multiple sclerosis and systemic lupus erythematosus. Arch Neurol.

[37] Naik H. (2022). Evaluating fatigue in patients recovering from COVID-19: validation of the fatigue severity scale and single item screening questions. Health Qual Life Outcomes.

[38] Cotler J., Holtzman C., Dudun C., Jason L.A. (2018). A brief questionnaire to assess post-exertional malaise. Diagnostics.

[39] Stevens S., Snell C., Stevens J., Keller B., VanNess J.M. (2018). Cardiopulmonary exercise test methodology for assessing exertion intolerance in myalgic encephalomyelitis/chronic fatigue syndrome. Front Pediatr.

[40] Nelson M.J. (2019). Diagnostic sensitivity of 2-day cardiopulmonary exercise testing in myalgic encephalomyelitis/chronic fatigue syndrome. J Transl Med.

[41] Borg G. (1998).

[42] Mahler D.A., Wells C.K. (1988). Evaluation of clinical methods for rating dyspnea. Chest.

[43] Yorke J., Moosavi S.H., Shuldham C., Jones P.W. (2010). Quantification of dyspnoea using descriptors: development and initial testing of the dyspnoea-12. Thorax.

[44] Daynes E., Gerlis C., Briggs-Price S., Jones P., Singh S.J. (2021). COPD assessment test for the evaluation of COVID-19 symptoms. Thorax.

[45] Boulding R., Stacey R., Niven R., Fowler S.J. (2016). Dysfunctional breathing: a review of the literature and proposal for classification. Eur. Respir. Rev..

[46] Gaffney A. (2024). Dysfunctional breathing after COVID-19: recognition and ramifications. Eur Respiratory Soc.

[47] Van Dixhoorn J., Duivenvoorden H. (1985). Efficacy of nijmegen questionnaire in recognition of the hyperventilation syndrome. J Psychosom Res.

[48] Van Dixhoorn J., Folgering H. (2015).

[49] Steinmann J. (2023). Validating the breathing vigilance questionnaire for use in dysfunctional breathing. Eur Respir J.

[50] Todd S. (2018). Novel assessment tool to detect breathing pattern disorder in patients with refractory asthma. Respirology.

[51] Pauwen N.Y. (2022). Validation criteria for PETCO2 kinetics during the hyperventilation provocation test in the diagnosis of idiopathic hyperventilation syndrome. J Clin Med.

[52] Nasreddine Z.S. (2005). The montreal cognitive assessment, MoCA: a brief screening tool for mild cognitive impairment. J Am Geriatr Soc.

[53] Alonso C.D., Matias-Guiu J.A. (2024). *Linking Neuroscience and Behavior in COVID-19*.

[54] Lynch S. (2022). Screening for brain fog: is the montreal cognitive assessment an effective screening tool for neurocognitive complaints post-COVID-19?. Gen Hosp Psychiatry.

[55] Reeves J.M. (2025). Effect of a 4-week pulmonary telerehabilitation program for people with respiratory post-acute sequelae of COVID-19–A randomised controlled trial. Eur J Physiother.

[56] Oliveira J.G.M. (2024). Assessment of the long-term physical capacity of COVID-19 survivors. Braz J Phys Ther.

[57] Freeman R. (2011). Consensus statement on the definition of orthostatic hypotension, neurally mediated syncope and the postural tachycardia syndrome. Auton. Neurosci..

[58] Dani M. (2021). Autonomic dysfunction in ‘long COVID’: rationale, physiology and management strategies. Clin Med.

[59] Raj S.R. (2021). Long-COVID postural tachycardia syndrome: an American autonomic society statement. Clin. Auton. Res..

[60] Ferreira Á.A. (2024). Applicability of heart rate variability for cardiac autonomic assessment in long-term COVID patients: a systematic review. J Electrocardiol.

[61] Suh H.-W., Kwon C.-Y., Lee B. (2023). Long-term impact of COVID-19 on heart rate variability: a systematic review of observational studies. Healthcare.

[62] Ghali A. (2023). The relevance of pacing strategies in managing symptoms of post-COVID-19 syndrome. J Transl Med.

[63] Sanal-Hayes N.E. (2023). A scoping review of ‘pacing’for management of myalgic encephalomyelitis/chronic fatigue syndrome (ME/CFS): lessons learned for the long COVID pandemic. J Transl Med.

[64] Dantas L.O., Barreto R.P.G., Ferreira C.H.J. (2020). Digital physical therapy in the COVID-19 pandemic. Braz J Phys Ther.

[65] Singh S.J. (2023). Balancing the value and risk of exercise-based therapy post-COVID-19: a narrative review. Eur. Respir. Rev..

[66] Daynes E., Gerlis C., Chaplin E., Gardiner N., Singh S.J. (2021). Early experiences of rehabilitation for individuals post-COVID to improve fatigue, breathlessness exercise capacity and cognition–A cohort study. Chron Respir Dis.

[67] Xia W. (2022). A telerehabilitation programme in post-discharge COVID-19 patients (TERECO): a randomised controlled trial. Thorax.

[68] McGregor G. (2024). Clinical effectiveness of an online supervised group physical and mental health rehabilitation programme for adults with post-covid-19 condition (REGAIN study): multicentre randomised controlled trial. BMJ.

[69] Daynes E. (2025). Post-hospitalisation COVID-19 rehabilitation (PHOSP-R): a randomised controlled trial of exercise-based rehabilitation. Eur Respir J.

[70] Meléndez-Oliva E. (2023). Efficacy of pulmonary rehabilitation in post-COVID-19: a systematic review and meta-analysis. Biomedicines.

[71] Pouliopoulou D.V. (2023). Rehabilitation interventions for physical capacity and quality of life in adults with post–COVID-19 condition: a systematic review and meta-analysis. JAMA Netw Open.

[72] Reeves J.M. (2024). Effect of a 4-week telerehabilitation program for people with post-COVID syndrome on physical function and symptoms: protocol for a randomized controlled trial. Phys Ther.

[73] Holland A.E. (2021). Defining modern pulmonary rehabilitation. An official American thoracic society workshop report. Ann Am Thorac Soc.

[74] Daynes E. (2024). Pulmonary rehabilitation for people with persistent symptoms after COVID-19. Chest.

[75] Daynes E. (2024). Changes in fatigue symptoms following an exercise-based rehabilitation programme for patients with long COVID. ERJ Open Res.

[76] Booth S., Johnson M.J. (2019). Improving the quality of life of people with advanced respiratory disease and severe breathlessness. Breathe.

[77] Enright S.J., Unnithan V.B., Heward C., Withnall L., Davies D.H. (2006). Effect of high-intensity inspiratory muscle training on lung volumes, diaphragm thickness, and exercise capacity in subjects who are healthy. Phys Ther.

[78] Beaumont M., Forget P., Couturaud F., Reychler G. (2018). Effects of inspiratory muscle training in COPD patients: a systematic review and meta-analysis. Clin Respir J.

[79] McCreery J.L., Mackintosh K.A., Mills-Bennett R., McNarry M.A. (2021). The effect of a high-intensity PrO2Fit inspiratory muscle training intervention on physiological and psychological health in adults with bronchiectasis: a mixed-methods study. Int J Environ Res Public Health.

[80] Severin R., Arena R., Lavie C.J., Bond S., Phillips S.A. (2020). Respiratory muscle performance screening for infectious disease management following COVID-19: a highly pressurized situation. Am J Med.

[81] McNarry M.A. (2022). Inspiratory muscle training enhances recovery post-COVID-19: a randomised controlled trial. Eur Respir J.

[82] Xavier D.M. (2024). Effects of respiratory muscular training in post-covid-19 patients: a systematic review and meta-analysis of randomized controlled trials. BMC Sports Sci Med Rehabil.

[83] Jimeno-Almazán A. (2022). Effects of a concurrent training, respiratory muscle exercise, and self-management recommendations on recovery from post-COVID-19 conditions: the RECOVE trial. J Appl Physiol.

[84] Abodonya A.M. (2021). Inspiratory muscle training for recovered COVID-19 patients after weaning from mechanical ventilation: a pilot control clinical study. Medicine (Baltimore).

[85] Gloeckl R. (2024). Practical recommendations for exercise training in patients with long COVID with or without post-exertional malaise: a best practice proposal. Sports Medicine-Open.

[86] Evans R., Pick A., Lardner R., Masey V., Smith N., Greenhalgh T. (2023). Breathing difficulties after covid-19: a guide for primary care.

[87] Durstenfeld M.S. (2022). Use of cardiopulmonary exercise testing to evaluate long COVID-19 symptoms in adults: a systematic review and meta-analysis. JAMA Netw Open.

[88] Genecand L. (2023). Dysfunctional breathing symptoms, functional impact and quality of life in patients with long COVID-19: a prospective case series. BMJ Open Respir Res.

[89] NHS England (2023). Commissioning guidance for Post COVID services for adults, children, and young people. https://www.england.nhs.uk/publication/national-commissioning-guidance-for-post-covid-services/.

[90] Spathis A. (2017). The breathing, thinking, functioning clinical model: a proposal to facilitate evidence-based breathlessness management in chronic respiratory disease. NPJ Prim Care Respir Med.

[91] Philip K.E. (2022). An online breathing and wellbeing programme (ENO Breathe) for people with persistent symptoms following COVID-19: a parallel-group, single-blind, randomised controlled trial. Lancet Respir Med.

[92] Thomas M. (2009). Breathing exercises for asthma: a randomised controlled trial. Thorax.

[93] Bruton A. (2018). Physiotherapy breathing retraining for asthma: a randomised controlled trial. Lancet Respir Med.

[94] Gall N.P., James S., Kavi L. (2022). Observational case series of postural tachycardia syndrome (PoTS) in post-COVID-19 patients. Br J Cardiol.

[95] Monaghan A. (2022). Orthostatic intolerance in adults reporting long COVID symptoms was not associated with postural orthostatic tachycardia syndrome. Front Physiol.

[96] Aranyó J. (2022). Inappropriate sinus tachycardia in post-COVID-19 syndrome. Sci Rep.

[97] Sheldon R.S. (2015). 2015 heart rhythm society expert consensus statement on the diagnosis and treatment of postural tachycardia syndrome, inappropriate sinus tachycardia, and vasovagal syncope. Heart Rhythm.

[98] Ormiston C.K., Świątkiewicz I., Taub P.R. (2022). Postural orthostatic tachycardia syndrome as a sequela of COVID-19. Heart Rhythm.

[99] Hira R. (2023). Objective hemodynamic cardiovascular autonomic abnormalities in post-acute sequelae of COVID-19. Can J Cardiol.

[100] Raj S.R. (2020). Canadian cardiovascular society position statement on postural orthostatic tachycardia syndrome (POTS) and related disorders of chronic orthostatic intolerance. Can J Cardiol.

[101] Putrino, D., Tabacof, L., Tosto-Mancuso, J., Wood, J., Cortes, M., Kontorovich, A., McCarthy, D., Breyman, E., Nasr, L., Neglia, A. and Duntz, J., 2021. Autonomic conditioning therapy reduces fatigue and improves global impression of change in individuals with post-acute COVID-19 syndrome.

[102] Fu Q., Levine B.D. (2018). Exercise and non-pharmacological treatment of POTS. Auton. neurosci..

[103] George S.A. (2016). The international POTS registry: evaluating the efficacy of an exercise training intervention in a community setting. Heart Rhythm.

[104] McGregor G. (2020). Protocol for a randomised controlled feasibility trial of exercise rehabilitation for people with postural tachycardia syndrome: the PULSE study. Pilot Feasibility Stud.

[105] Quinn K.L. (2023). Cardiovascular considerations in the management of people with suspected long COVID. Can J Cardiol.

[106] World Health Organization (WHO) Clinical management of COVID-19: living guideline, 15 September 2022. (World health organization, 2022). 35917394

[107] MacKinnon C. (2025). Perspectives of rehabilitation professionals on long COVID interventions to facilitate return-to-work. Can. J. Occup. Ther..

[108] DeMars J. (2022). Recommendations for employers, insurers, human resource professionals on return to work for people living with long COVID. Realize Canada.

[109] World Health Organisation (WHO). Post COVID-19 condition (long COVID). (https://www.who.int/news-room/fact-sheets/detail/post-covid-19-condition-(long-covid)).

[110] Canadian Guidelines for Post COVID-19 Condition (CAN-PCC). McMaster University & Cochrane Canada; 2025. hhttps://canpcc.ca.

[111] Su Y. (2022). Multiple early factors anticipate post-acute COVID-19 sequelae. Cell.

[112] Klein J. (2023). Distinguishing features of long COVID identified through immune profiling. Nature.

[113] Yeoh Y.K. (2021). Gut microbiota composition reflects disease severity and dysfunctional immune responses in patients with COVID-19. Gut.

[114] Liu Q. (2022). Gut microbiota dynamics in a prospective cohort of patients with post-acute COVID-19 syndrome. Gut.

[115] Proal A.D., VanElzakker M.B. (2021). Long COVID or post-acute sequelae of COVID-19 (PASC): an overview of biological factors that may contribute to persistent symptoms. Front Microbiol.

[116] Klok F.A. (2020). Incidence of thrombotic complications in critically ill ICU patients with COVID-19. Thromb Res.

[117] Cui S., Chen S., Li X., Liu S., Wang F. (2020). Prevalence of venous thromboembolism in patients with severe novel coronavirus pneumonia. J Thromb Haemost.

[118] Nalbandian A. (2021). Post-acute COVID-19 syndrome. Nat Med.

[119] Goshua G. (2020). Endotheliopathy in COVID-19-associated coagulopathy: evidence from a single-centre, cross-sectional study. Lancet Haematol.

[120] Ackermann M. (2020). Pulmonary vascular endothelialitis, thrombosis, and angiogenesis in Covid-19. N Engl J Med.

[121] Shan Z.Y. (2020). Neuroimaging characteristics of myalgic encephalomyelitis/chronic fatigue syndrome (ME/CFS): a systematic review. J Transl Med.

